# KCa3.1 and TRPM7 Channels at the Uropod Regulate Migration of Activated Human T Cells

**DOI:** 10.1371/journal.pone.0043859

**Published:** 2012-08-27

**Authors:** Zerrin Kuras, Yeo-Heung Yun, Ameet A. Chimote, Lisa Neumeier, Laura Conforti

**Affiliations:** 1 Department of Internal Medicine, University of Cincinnati, Cincinnati, Ohio, United States of America; 2 Department of Molecular and Cellular Physiology, University of Cincinnati, Cincinnati, Ohio, United States of America; 3 Department of Bioengineering, North Carolina Agricultural and Technical State University, Greensboro, North Carolina, United States of America; Beth Israel Deaconess Medical Center, United States of America

## Abstract

The migration of T lymphocytes is an essential part of the adaptive immune response as T cells circulate around the body to carry out immune surveillance. During the migration process T cells polarize, forming a leading edge at the cell front and a uropod at the cell rear. Our interest was in studying the involvement of ion channels in the migration of activated human T lymphocytes as they modulate intracellular Ca^2+^ levels. Ca^2+^ is a key regulator of cellular motility. To this purpose, we created protein surfaces made of the bio-polymer PNMP and coated with ICAM-1, ligand of LFA-1. The LFA-1 and ICAM-1 interaction facilitates T cell movement from blood into tissues and it is critical in immune surveillance and inflammation. Activated human T lymphocytes polarized and migrated on ICAM-1 surfaces by random walk with a mean velocity of ∼6 µm/min. Confocal microscopy indicated that Kv1.3, CRAC, and TRPM4 channels positioned in the leading-edge, whereas KCa3.1 and TRPM7 channels accumulated in the uropod. The localization of KCa3.1 and TRPM7 at the uropod was associated with oscillations in intracellular Ca^2+^ levels that we measured in this cell compartment. Further studies with blockers against Kv1.3 (ShK), KCa3.1 (TRAM-34), CRAC (SKF-96365), TRPM7 (2-APB), and TRPM4 (glibenclamide) indicated that blockade of KCa3.1 and TRPM7, and not Kv1.3, CRAC or TRPM4, inhibits the T cell migration. The involvement of TRPM7 in cell migration was confirmed with siRNAs against TRPM7. Downregulation of TRPM7 significantly reduced the number of migrating T cells and the mean velocity of the migrating T cells. These results indicate that KCa3.1 and TRPM7 selectively localize at the uropod of migrating T lymphocytes and are key components of the T cell migration machinery.

## Introduction

The capability of T lymphocytes to migrate is a crucial part of the adaptive immune response. T cells migrate continuously in and out of the bloodstream to lymphoid organs and to tissues in search of specific antigens to carry out immune surveillance [1], [2].

During migration T cells acquire an asymmetric morphology and polarize, forming in the front part of the cell lamellipodia, whose leading-edge is projected towards the migration trajectory, followed by a trailing uropod [3], [4]. The leading-edge contains chemokine receptors, like C-X-C chemokine receptor type 4 (CXCR-4), T cell receptor (TCR) and filamentous actin (F-actin) [3], [4]. These allow sensing of chemoattractants, adhesion and F-actin polymerization to drive forward movement [4]. Other proteins, including the adhesion proteins intercellular adhesion molecule-1 (ICAM-1) and CD44, together with ERM proteins (ezrin, radixin and moesin) and the mitochondria, instead polarize to the uropod, which undergoes cycles of attachment, release and retraction, thus completing the crawling process that allows cell motility [3], [4].

Many factors are known to be important for cell migration including the cytoskeleton and integrins. In particular, the interaction between LFA-1 and its ligand ICAM-1 facilitates T cell interaction with the vascular endothelium and extravasation from blood into tissues and it is critical in immune surveillance and inflammation [4]. The interaction of LFA-1 with ICAM-1 induces LFA-1 activation which is followed by signal cascades, resulting in F-actin polymerization [4].

Ion channels have recently emerged as key players in cell motility as they preside over the cell volume and Ca^2+^ homeostasis [5]. Changes in cell shape occur during cell movement which could be viewed as intermittent local swelling and shrinkage. Furthermore, the intracellular Ca^2+^ concentration ([Ca^2+^]_i_) controls various aspects of cell migration including the F-actin polymerization/depolymerization necessary for the propulsion of the cell [4], [5].

We were interested in the role of ion channels in T cell migration. Two potassium channels are expressed in human T cells, the voltage-gated K^+^ channel Kv1.3 and the Ca^2+^-activated K^+^ channel KCa3.1 (also known as IKCa1) [6], [7]. These K^+^ channels regulate membrane potential and maintain the electrochemical gradient necessary for Ca^2+^ influx [8]. Expression of these channels depends on the T cell activation state with a predominance of Kv1.3 in chronically activated effector memory (T_EM_) cells, while KCa3.1 is highly expressed in activated naïve and central memory (T_CM_) cells [8], [9]. Both K^+^ channels have been implicated in the mobility of various cell types. KCa3.1 regulates cell migration in human lung mast cells and cancer cells among others [5]. Kv1.3 regulates the migration velocity of microglia and vascular smooth muscle cells [5]. Furthermore, Kv1.3 regulates migration of T_EM_ cells in-vivo [10].

The main Ca^2+^ channel in human T cells is the Ca^2+^-release activated Ca^2+^ channel (CRAC) which has been described as part of the migration machinery of vascular smooth muscle and dendritic cells [11], [12]. Also the transient receptor potential melastatin 7 (TRPM7), a Ca^2+^- and Mg^2+^-permeant ion channel expressed in T cells, has been implicated in the migration of fibroblasts and tumor cells [13], [14], [15]. Although ion channels play such important roles in cell migration, the contribution of K^+^ and Ca^2+^ channels in T cell motility and their localization in a migrating T cell are not understood.

The current study was undertaken to investigate the distribution of ion channels in polarized migrating T cells and to establish whether specific K^+^ and Ca^2+^ channels regulate the migration of activated T cells. Using artificial ICAM-1 surfaces, we demonstrate the selective involvement of KCa3.1 and TRPM7 channels at the uropod in the migration of activated human T cells [16].

## Methods

### Cells

The study was approved by the University of Cincinnati Institutional Review Board. Primary human CD3^+^ T cells were isolated by E-rosetting (StemCell Tech., Vancouver, Canada) from venous blood from consenting healthy donors followed by Ficoll-Paque density gradient centrifugation (ICN Biomedicals, Aurora, OH, USA) [17]. The isolated T cells were cultured in RPMI medium supplemented with 10% pooled male human AB serum (Intergen, Milford, MA, USA), 200 U ml^−1^ penicillin, 200 µg ml^−1^ streptomycin and 10 mM Hepes [18]. T cell activation was achieved by stimulation with 4 µg ml^−1^ phytohemaglutinin (PHA, Sigma-Aldrich, St. Louis, MO, USA) in the presence of peripheral blood mononuclear cells (PBMC) for 72 hr. Blood was obtained from either healthy volunteers or healthy blood bank donors (unutilized blood units from the Hoxworth Blood Bank Center, Cincinnati, OH, USA). Written informed consent was obtained from the healthy volunteers.

### Transfection

A GFP-tagged Kv1.3 (pEGFP-Kv1.3; kind gift of Dr. Takimoto, Department of Bioengineering, Nagaoka University of Technology, Nagaoka, Niigata, Japan), and a YFP-tagged KCa3.1 construct (pEYFP-KCa3.1) and a hemagglutinin (HA)- tagged KCa3.1 construct with the HA tag present in the extracellular loop between the S3 and S4 domains (HA-KCa3.1; both KCa3.1 constructs kind gifts from Dr. Devor, Department of Cell Biology and Physiology, University of Pittsburgh) were used for transfection [17], [19], [20]. The TRPM7 siRNA (a kit of 3 unique siRNA duplexes) and the scrambled negative control siRNA (scrRNA) were obtained from OriGene Technologies (Rockville, MD, USA). Activated T cells were transiently transfected using the Amaxa Nucleofector technology (Lonza, Cologne, Germany). The transfection was done using 10*10^6^ T cells, 5 µg DNA or 10 nM siRNA and program U-14, according to the manufacturer’s instructions. For the siRNA experiments, cells were cotransfected with pMAX GFP supplied in the Amaxa Nucleofector kit at a GFP:siRNA ratio of 1∶10 as previously described [21], [22]. GFP expression was used to visualize transfected cells both in patch-clamp and live microscopy experiments.

### Electrophysiology

Patch-clamp experiments were performed using Axopatch 200B amplifier (Axon Instruments, Foster City, CA, USA) in whole cell configuration as previously described. Kv1.3 currents were recorded with an external solution of the following composition (in mM): 150 NaCl, 5 KCl, 2.5 CaCl_2_, 1.0 MgCl_2_, 10 glucose and 10 HEPES, pH 7.4, 340 mOsm. The pipette solution was composed of (mM): 134 KCl, 1 CaCl_2_, 2 MgCl_2_, 10 EGTA, and 10 HEPES, pH 7.4, 330 mOsm [23]. The Kv1.3 currents were measured by depolarizing voltage steps to +50 mV from a holding potential (HP) of −80 mV every 30 s. The external solution for KCa3.1 currents were (in mM): 160 Na^+^ aspartate (used to reduce chloride “leak” currents [24]), 4.5 KCl, 2 CaCl_2_, 1 MgCl_2_, and 10 HEPES, pH 7.4, 310 mOsm. The pipette solution was (in mM): 145 K^+^ aspartate, 8.5 CaCl_2_, 2 MgCl_2_, 10 EGTA and 10 HEPES, pH 7.2, 318 mOsm, with an estimated free Ca^2+^ concentration of 1 µM [25]. The KCa3.1 currents were induced by ramp depolarization from −120 mV to +40 mV from a HP of −80 mV every 10 s. KCa3.1 current amplitude was measured at +40 mV. TRPM7 currents were recorded in the following external solution (in mM): 135 CH_3_SO_3_Na, 5 CsCl, 1 CaCl_2_, 10 Hepes, pH 7.4, 280 mOsm. The pipette solution contained (in mM): 120 CH_3_O_3_SCs, 5 CsCl, 3.1 CaCl_2_, 10 BAPTA, 10 Hepes, pH 7.2, 288 mOsm, with an estimated free Ca^2+^ concentration of 100 nM [26]. The TRPM7 currents were elicited by ramp depolarization from −100 mV to +100 mV from a HP of 0 mV every 10 s. The digitized signals were stored and analyzed using pClamp 9 software (Axon Instruments, Foster City, CA, USA). Experiments were conducted at room temperature (22°C).

### Fabrication of ICAM-1 Surfaces

**Figure 1:**
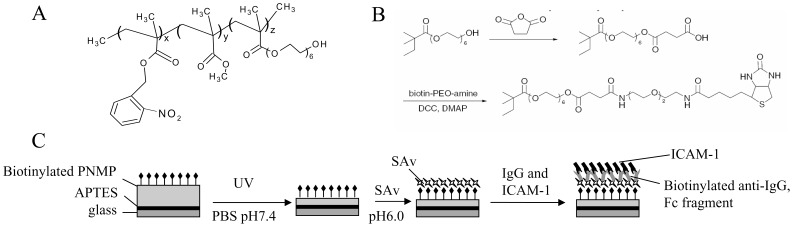
Fabrication of protein surface with intercellular adhesion molecule-1 (ICAM-1). A. Structure of the terpolymer PNMP. B. Biotinylation of PNMP. The treatment of PNMP with succinic anhydride resulted in a carboxyl group which is then biotinylated by biotin-PEO-amine. The biotinylation allows a more specific protein binding compared to the binding of a carboxyl group. C. Diagram of protein patterning on PNMP. A glass coverslip is coated with a cationic layer of APTES and on top of this a layer of PNMP is deposited by spin-coating. The appropriate thickness of PNMP is achieved by UV irradiation and washing in pH 7.4 PBS. For the optimization of the thickness of the surfaces used in our experiments refer to the supplemental data shown in Table S1 which reports the experimental conditions necessary to achieve the appropriate PNMP thickness (200 nm). After these steps, streptavidin, which binds to the biotin, is added followed by addition of the biotinylated IgG and ICAM-1.

ICAM-1 surfaces were generated by deposition of human ICAM-1 on the biocompatible random terpolymer poly(o-nitrobenzyl methacrylate-r-methyl methacrylate-r-poly(ethylene glycol) methacrylate) (PNMP). The PNMP polymer allows great flexibility and the possibility of depositing multiple proteins in a specific geometry via photolithography [16]. This same polymer will eventually allow us to fabricate antigen presenting cell arrays with incorporated activation sites (circles of antibodies against the T cell receptor) to study how the membrane distribution of channels changes going from a moving T cell to a cell that has made contact with an activation site. PNMP has two advances over other photoresistent polymers: it is soluble in a physiological pH which allows deposition of proteins, and all deep UV irradiation required for solubility can be done before the proteins get attached to the surface [16]. PNMP was synthesized by free-radical polymerization of the following monomers: O-nitrobenzyl methacrylate, methyl methacrylate and poly(ethylene glycol) methacrylate (Fig. 1A) [16]. All the reagents used in the fabrication of ICAM surfaces were from Sigma Aldrich (St. Louis, MO, USA) unless otherwise specified. The synthesis took place in a solvent of ethyl acetate (Pharmco Products Inc., Brookfield, CT, USA) and was initiated by 2,2′-Azobis(2-methylproionitrile). The reaction was completed by 4-methoxyphenol and the polymer was purified twice by precipitation in diethyl ether. The obtained PNMP was then biotinylated to allow protein immobilization (Fig. 1B). The biotinylation was initiated by dissolving the PNMP polymer with succinic anhydride in anhydrous dichloroethane and adding N-methylimidazole drop wise for 15 hours with stirring. This procedure results in a carboxyl group at the end of the poly(ethylene glycol) methacrylate monomer. The final step for the biotinylation was achieved by mixing the carboxylated PNMP polymer with biotin-PEO-amine (Pierce Biotechnology, Rockford, IL, USA), 4-dimethylaminopyridine and dichloromethane, then adding a solution of N, N’-dicyclohexylcarbodiimide in dichloromethane drop wise while stirring for 18 hours. The final product was afterward purified by precipitation in n-hexane (Tedia Company, Fairfield, OH, USA). The PNMP polymer must be carboxylated and biotinylated before the UV exposure since the polymer develops a pH-sensitivity after UV irradiation by cleaving off the o-nitrobenzyl group [27].

The synthetic surface was fabricated as followed (Fig. 1C). The glass surface was conjugated with 3-aminopropyltriethoxysilane (APTES) by covalent binding to the etched surface of the glass coverslip which became cationic after placing in water. PNMP was spun-coat onto a designated surface to guarantee a thin and uniform layer suitable for UV irradiation, even development, and eventually protein immobilization. The process of PNMP coating was optimized with Si/SiO_2_ wafer using a Chemat Technology Spin-Coater Model # KW-4A. Optimum spin specifications for uniform layering to certain thicknesses were determined for each concentration of PNMP solution by KLA Profilometer (Table S1). After the optimization of the spin-coating, the UV irradiation time and pH sensitivity was optimized by using a UV mask of Transmission electron microscopy (TEM) grids. With an increasing time from 20 to 60 min of UV irradiation the grid patterning got worse which demonstrated that the optimum irradiation time was 20 min (Fig. S1A). Furthermore, the thickness of the PNMP layer can be reduced by taking advantage of its unique pH sensitivity after UV exposure. The PNMP polymer is only slightly soluble in PBS at pH 6.0, whereas its solubility is increased as the pH levels becomes more basic and hits a maximum around the physiological pH level of 7.4 as indicated by the amount of PNMP thickness removed (Fig. S1B, C). The obtained minimum thickness of the polymer layer allowed optical imaging of the prepared cells. Overall, these optimization indicated an optimal experimental PNMP photolithography conditions with a 20 min UV irradiation at 225 nm and pH 6.5–7.0 PBS as a developer, as indicated by the optimal grid definition achieved at these conditions (Fig. S1D).

Once the biotinylated PNMP polymer was spin-coated onto the APTES conjugated glass surface, UV irradiated and washed with pH 7.4 PBS to get a thin layer of 200 nm, streptavidin was attached by incubation in pH 6.0 PBS for 40 min at room temperature (Fig. 1C). Subsequently, the surface was washed in pH 6.0 PBS followed by the deposit of the proteins: biotinylated anti-IgG/Fc fragment and finally ICAM-1.

### Cell Preparation and Live Cell Microscopy

Activated T cells were plated on ICAM-1 protein surfaces at a final concentration of 6–10*10^6^ ml^−1^ in a solution of the following composition (in mM): 155 NaCl, 4.5 KCl, 1 MgCl_2_, 2 CaCl_2_, 10 HEPES, and 10 glucose, pH 7.4, 323±2 mOsm (n = 3). Cells were maintained at 37°C for 2 hr before starting the migration velocity experiments. After this time, the arrays were transferred to a heated microscopy chamber (35.0±0.1°C; n = 153). Time-lapse microscopy was performed using the InCytIm3XMTD imaging system (Intracellular Imaging, Cincinnati, OH, USA) and bright field images were acquired at the rate of 20 images min^−1^. The analysis and cell tracking was performed using MetaMorph software (Molecular Devices, Sunnyvale, CA, USA).

The criteria for analysis was defined as follows: T cells that acquired leading edge and trailing uropod were considered as “polarized” and were categorized as “polarized motile” if they were able to move away from their initial position with a minimum mean velocity of 1.5 µm/min. Polarized T cells that moved around a fixed contact point (i.e. did not travel any significant distance) were defined as “tethers”. Cells that remained at the same coordinate were defined as “arrests”. Only “polarized motile” cells were considered as migrating cells and included in our analysis.

### Measurements of Intracellular Ca^2+^

Cells were plated on ICAM-1 protein surfaces as described above and maintained at 37°C for 2 hr. Cells were loaded with 1 µM Fura-2/AM as previously described and the experiments were performed using InCytIm3XMTD imaging system (Intracellular Imaging, Cincinnati, OH, USA) on a heated microscopy chamber (35.3±0.2°C; n = 7) [18]. Experiments were conducted in the same solution used for the motility experiments. The Ca^2+^-free solutions had the following composition (in mM): 155 NaCl, 4.5 KCl, 1 MgCl_2_, 10 HEPES, 10 glucose, and 2 EGTA, pH 7.4. The ratio between the wavelength of 340 and 380 nm was calculated from migrating T cells in MetaMorph software (Molecular Devices, Sunnyvale, CA, USA). An increase in the 340/380 ratio ≥0.1 ratio units (r.u.) was previously described as a significant increase in [Ca^2+^]_i_ [28]. Ca^2+^ oscillations were defined by the presence of ≥2 peak in the fluorescence ratio during a recording time of at least 2 min.

### Immunohistochemistry

T cells were pre-incubated on ICAM-1 protein surface for 2 hr at 37°C to allow the cells to migrate, then fixed with 3.7% formaldehyde in PBS and blocked using FBS. Protein staining was performed by incubating the cells with primary antibodies either with or without permeabilization by 0.1% Triton X-100 depending whether the antibody was against an intracellular or extracellular epitope. The following antibodies were used: mouse anti-CD44 (R&D Systems, #BBA10), mouse anti-CXCR-4 (R&D Systems, #FAB172B), rabbit anti-HA (Sigma Aldrich #H6908), rabbit extracellular anti-Kv1.3 (Millipore, #AB5589), rabbit extracellular anti-Orai1 (Alomone labs, #ACC-060), rabbit intracellular anti-TRPM4 (Aviva Systems Biology, #ARP35268_P050), and goat intracellular anti-TRPM7 (Abcam, #ab729). The specificity of the extracellular anti-Kv1.3 antibody has been shown by us [21], [28]. The anti-TRPM7 goat-polyclonal antibody we used was previously used by Chen et al. [14]. In this paper the authors have performed Western blot assays and shown that this antibody recognizes a band of the expected molecular weight which is downregulated by TRPM7 siRNAs. Similarly, the antibody against Orai1 that we used was used by Bessialon et al. [29]. In this manuscript they showed that the antibody recognizes a band of the expected MW which is downregulated by specific siRNAs against Orai1. Furthermore, they compared this antibody to others commercially available. Similar results were obtained using anti-Orai1 antibodies from Sigma (catalog no. O8264) and ProSci (catalog no. 4041), confirming the validity of the Alomone antibody. The specificity of anti-Orai1, anti-TRPM4, and anti-TRPM7 antibodies was further confirmed by preadsorption to the corresponding antigens (Fig. S2). These Orai1 and TRPM7 antibodies were used in other studies to determine the distribution of the corresponding channels [14], [30], [31]. Fixed cells were exposed to primary antibodies overnight at 4°C followed by secondary antibodies (Invitrogen Corp., Eugene, OR, USA). Cells were visualized by confocal microscopy (Axioscope microscope or Zeiss LSM 510 or 710 microscopes, Carl Zeiss MicroImaging LLC, Thornwood, NY, USA) using a 100X oil objective lens or Zeiss-Apochromat 63X.

Data were obtained and analyzed by LSM Image Examiner or Zen 2010 (Carl Zeiss MicroImaging LLC). The image threshold and background were adjusted and the colocalization was measured using the Pearson’s correlation coefficient on the raw images after designating the entire cell as region of interest (ROI), and was determined using following equation in the LSM Image Examiner (Carl Zeiss MicroImaging LLC):
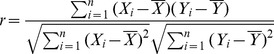
(1)Where *r* is the Pearson’s correlation coefficient *X_i_* and *Y_i_*, are the intensities of the individual pixels of red and green channels, respectively within the ROI, whereas 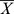 and 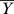 are the average intensities for the red and green channels [21], [32], [33]. The values for *r* range from −1 to +1, where +1 indicates strong correlation, and −1 indicates no correlation [33]. Only cells which achieved our inclusion criteria (asymmetric and polarized cell morphology) were quantified. Cells with round morphology were excluded from the analysis. The cells displayed diverse morphologies due to cell to cell variability and differences in the migration stage.

### RT-qPCR

Total RNA was isolated from primary activated T cells using E.Z.N.A.™ Total RNA Isolation System (Omega Bio-Tek, Norcross GA) according to the manufacturer’s instructions. cDNA was synthesized from 500 ng of RNA using TaqMan® Reverse Transcription Reagents (Applied Biosystems) as per the manufacturer’s instructions. TaqMan probe based RT-qPCR was performed for TRPM7. Predesigned Assay-on-Demand™ Primers for TRPM7 and glyceraldehyde 3-phosphate dehydrogenase (GAPDH, internal control) were obtained from Applied Biosystems (Life Science Technologies, Carlsbad CA). The reaction was set up in a 48-well plate by adding 40 ng cDNA, 1x TaqMan® Fast Universal PCR Master Mix (Applied Biosystems) and 1 µl of Assay-on-Demand™ Primers, as described by us previously [21]. A SYBR® Green RT-qPCR was performed for Orai1 and TRPM4 as previously described [22]. The primers for Orai1 and TRPM4 had the following sequences: Orai1 forward: CTT CAG TGC CTG CAC CAC AG, Orai1 reverse: CCT GGA ACT GTC GGT CAG TC; TRPM4 forward: TCG GCA AAG TAC AGG GCA AC and TRPM4 reverse: AGG CGC AAG TGG GAG ATG AC. GAPDH was used as internal control [22]. Orai1, TRPM4 and GAPDH primers were purchased from Integrated DNA Technologies (Coralville IA). Briefly, 40 ng cDNA, 1x Fast SYBR® Green Master Mix (Applied Biosystems) and 1 µg of the sense and anti-sense primers were added to a 96-well plate in quadruplicate. RT-qPCR reaction was cycled in Applied Biosystems StepOne™ Real-time PCR system (Applied Biosystems). The reactions were cycled under the following conditions: 95°C for 20 seconds, followed by 40 cycles at 95°C for 3 seconds and 60°C for 30 seconds. Melting curves were performed to verify the specificity of the product. All samples were cycled in quadruplicate. C_T_ values were measured using StepOne software v2.1 (Applied Biosystems). C_T_ values for the genes of interest were normalized against measured C_T_ values for GAPDH and the ΔΔ C_T_ values were calculated. Relative Quantity (RQ) values were calculated as the 2^−ΔΔ CT^ values which represented the fold change in gene expression as compared to controls [21], [22].

### Chemicals

ShK was obtained from Bachem Americas, Inc. (Torrance, CA, USA), TRAM-34 was a kind gift from Dr. Wulff (Department of Pharmacology, UC Davis, CA, USA). SKF-96365 hydrochloride was purchased from Calbiochem (EMD Biosciences, Inc., La Jolla, CA, USA), glibenclamide from Tocris Bioscience (Ellisvilee, MO, USA), and 2-aminoethyl diphenyl borate (2-APB) was obtained from Sigma-Aldrich.

### Data Analysis

All data are presented as means ± SEM for n≥3. Statistical analyses were performed using Student’s t-test (paired or unpaired). One-way ANOVA was performed as described in the figure legends using SPSS Sigma Stat 3.0 software; p<0.05 was defined as significant.

## Results

### Activated CD3^+^ T Cells Migrate on ICAM-1 Protein Surfaces

**Figure 2:**
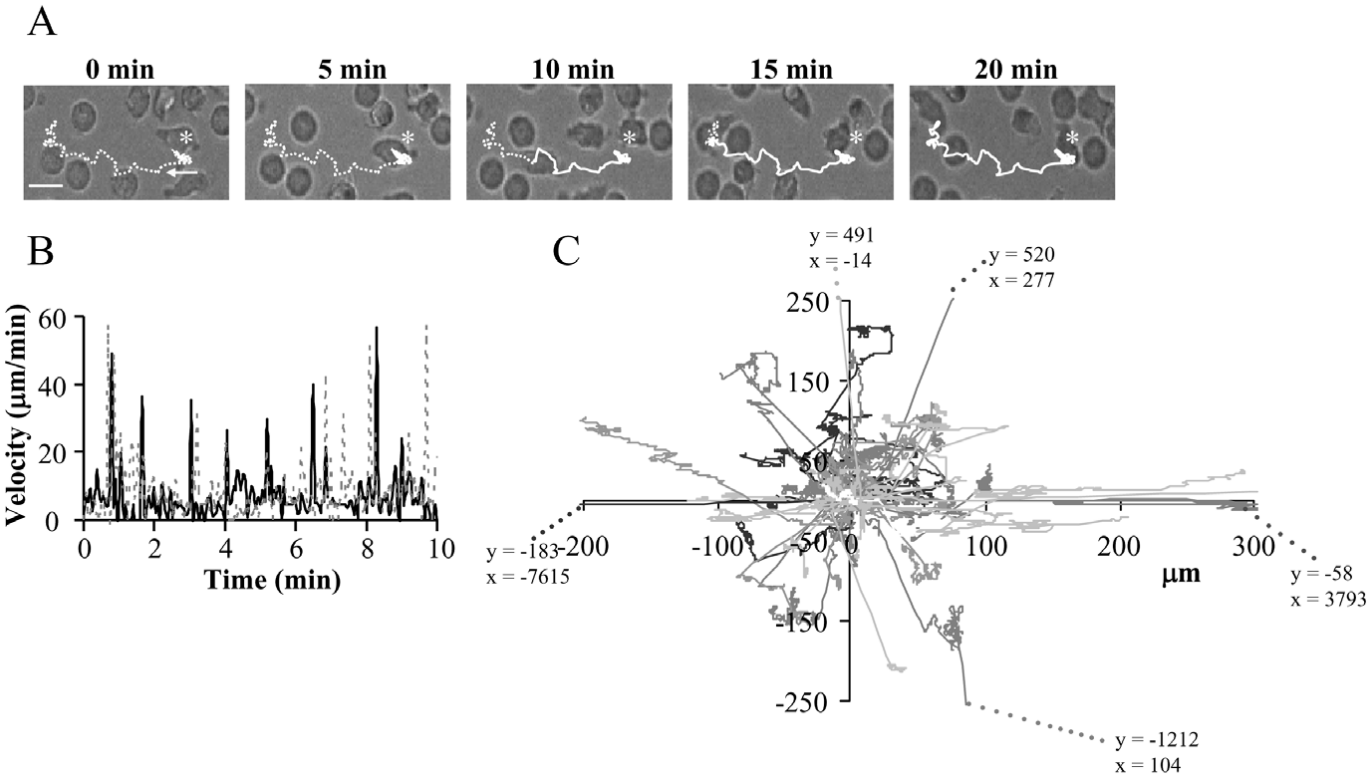
Movement of human activated CD3^+^ T cells on ICAM-1 surfaces by random walk. A. Representative time-lapse bright-field microscopy of an activated T cell migrating on an ICAM-1 surface. Asterisks indicate the starting point of the cell of interest, and the arrow shows the direction of the movement. The dotted line represents the coming track whereas the continuous line is the covered distance. Scale bar  = 5 µm. B. Time-dependent distribution of the migration velocity of two representative activated T cells each defined by either a continuous or dotted line. C. Tracks of 54 migrating T cells on ICAM-1 normalized to their starting coordinates. The x-y coordinates were determined in MetaMorph software. The final x–y values reached by 5 cells are out of the x/y axis ranges. The corresponding x–y coordinates are given for each cell at the end of the extended dotted lines.

The migration of activated primary human CD3^+^ T cells was studied on ICAM-1 surfaces. The ICAM-1 surfaces were generated by deposition of human ICAM-1 proteins on the biocompatible polymer PNMP [16]. Details on the preparation and characterization of the ICAM-1 surfaces are reported in the method section and as supplemental material. The average percentage of migrating untreated cells was 19±1% (n = 29 experiments from 9 donors, with 260±14 cells/experiment) ranging from 10% to 42%. Activated T cells migrated with a mean velocity of 5. 0±0.1 µm/min (n = 198 from 12 independent donors; mean ± SEM), with a minimum velocity of 1.5 µm/min, maximum velocity of 12.7 µm/min and a median velocity of 5.1 µm/min (Fig. 2A–B), which was comparable to earlier in-vitro studies [34]. The distribution of migration velocities for two representative T cells is shown in Fig. 2B. The velocities varied from 0.5 µm/min to more than 40 µm/min, representing a stop-and-go migration pattern. Furthermore, T cell migration occurred by amoeboid random walk (Fig. 2C). This kind of migratory behavior was observed previously in in-vivo studies in the lymph nodes [10]. Thus, these surfaces are useful tools for studying the mechanisms mediating T lymphocyte motility as they preserve the in-vivo motility of T cells.

Ion channels are known regulators of cell motility and both K^+^ channels expressed in human T lymphocytes, Kv1.3 and KCa3.1, have been implicated in the motility of different cell types [5]. We thus conducted experiments to determine whether a specific K^+^ channel regulates the migration of activated human T cells.

### KCa3.1, but not Kv1.3, Channels Control the Migration of Activated Human T Cells

**Figure 3:**
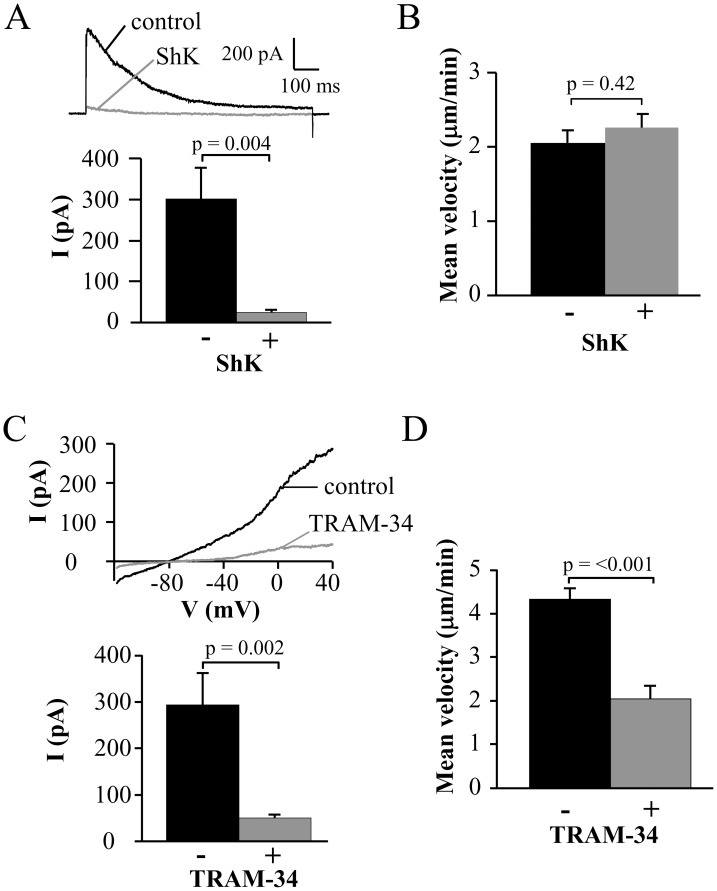
Selective role of KCa3.1 on the migration of activated CD3^+^ T cells. A. ShK inhibition of native Kv1.3 currents. Representative Kv1.3 currents were recorded in activated T cells before (control) and after extracellular application of 10 nM ShK. Currents were elicited by depolarizing pulses to +50 mV from a holding potential (HP) of −80 mV every 30 s. The average inhibition of Kv1.3 currents is shown in the lower panel (n = 6). B. Mean velocity before and after ShK (n = 7). The effect of the Kv1.3 inhibitor ShK (10 nM) on cell migration was determined by following single cells for 20 min before and after treatment with the blocker. C. TRAM-34 inhibition of native KCa3.1 currents. Representative KCa3.1 currents were recorded in activated T cells before (control) and after extracellular application of 250 nM TRAM-34. Currents were induced from a HP of −80 mV by a ramp depolarization from −120 mV to +40 mV every 10 s. The average KCa3.1 currents in absence and presence of TRAM-34 are shown in the lower panel (n = 9). D. Mean velocity before and after TRAM-34 (n = 22). The effect of 250 nM TRAM-34 was determined by following single cells for 20 min before and after TRAM-34 application.

To determine the K^+^ channels involved in activated T cell migration we used selective blockers of Kv1.3 and KCa3.1. Activated CD3^+^ T cells had an average capacitance of 4.6±0.2 pF (n = 32). The average Kv1.3 current amplitude was 452±57 pA (n = 12, from four independent healthy donors), giving 316±40 channels/cell (considering a conductance of 11 pS) which is comparable with the values reported in the literature for activated T cells [35]. The specific blocker ShK (10 nM) was used to inhibit Kv1.3 [36]. The efficacy of this blocker is shown in Fig. 3A. Kv1.3 currents, which display the characteristic C-type inactivation with an inactivation time constant of 348.1±72.1 ms (n = 6), were fully inhibited by 10 nM ShK [37], [38]. This concentration of ShK failed to inhibit the T cell migration (Fig. 3B). The mean velocity of single activated T cells was measured for 20 min before and after application of ShK (Fig. S3A). Similar velocities were measured before and after ShK application (Fig. 3B). These results indicated that Kv1.3 was not involved in T cell migration.

Similar experiments were performed to investigate the role of KCa3.1 in T cell migration. We used the specific inhibitor of KCa3.1 TRAM-34 [39]. Electrophysiological experiments confirmed that KCa3.1 activity was blocked by 250 nM TRAM-34 (Fig. 3C). The same concentration of TRAM-34 inhibited T cell migration (Fig. 3D and Fig. S3B). While there was no change in the percentage migrating cells after TRAM-34 application (28±9% with TRAM-34 and 35±9% before TRAM-34, out of a total 454–557 cells analyzed from 3 arrays and 2 donors, p = 0.65), TRAM-34 reduced the migration velocity. The mean baseline velocity of 4.3±0.3 µm/min decreased significantly to 2.0±0.3 µm/min after TRAM-34 application (n = 22, 2 donors; Fig. 3D). Hence, our measurements indicated a significant role of KCa3.1 in T cell migration.

Migrating cells are polarized into the leading-edge in the cell front and the uropod in the cell rear [3], [4]. Experiments were thus conducted to determine whether these functionally distinct K^+^ channels are also differentially compartmentalized in moving cells.

### K_Ca_3.1 is Located in the Uropod Whereas Kv1.3 Polarizes to the Leading-edge

**Figure 4:**
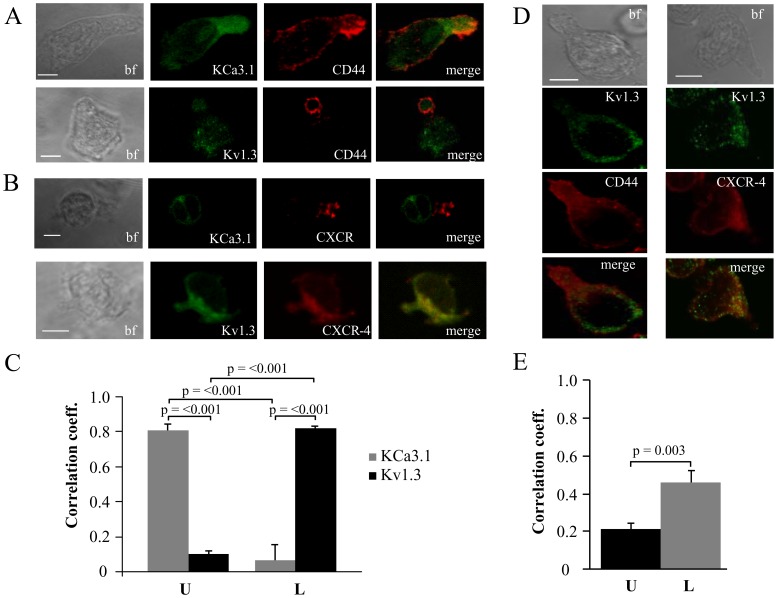
Differential localization of Kv1.3 and KCa3.1 in migrating T cells. A. Distribution of Kv1.3 and KCa3.1 at the uropod. T cells were transiently transfected with either YFP-KCa3.1 or GFP-Kv1.3 (green) and stained with anti-CD44 antibody (uropod; red) without permeabilization. Yellow areas in the merge images indicate colocalization. Scale bar  = 5 µm. B. Distribution of Kv1.3 and KCa3.1 at the leading-edge. T cells, transfected with either YFP-KCa3.1 or GFP-Kv1.3 (green) and stained with anti-CXCR-4 antibody (leading-edge; red) without permeabilization, were analyzed by confocal microscopy. Colocalization between the two proteins is indicated by yellow areas in the merge images. Scale bar  = 5 µm. C. Correlation coefficients for KCa3.1 and Kv1.3 localization in the uropod (U) and leading-edge (L). The data are the average of n = 15 cells for KCa3.1 at the U and n = 8 at the L, and n = 16 for Kv1.3 at the U and n = 11 at the U from 2 healthy individuals. Statistical significance was established by one way ANOVA. D. Localization of native Kv1.3 in the leading-edge. T cells from one healthy individual were fixed and stained with extracellular anti-Kv1.3 antibody (green) together with antibodies either against CD44 (red; left) or CXCR-4 (red, right). Yellow colors in the merge images indicate strong correlation. Scale bar  = 5 µm. E. Average Correlation coefficients of native Kv1.3 with the leading-edge (L) (n = 9) and the uropod (U) markers (n = 11).

The distribution of Kv1.3 and KCa3.1 in moving cells was established in confocal microscopy experiments by determining their degree of colocalization with markers of the leading-edge and the uropod. Due to the lack of commercially available antibodies specific for KCa3.1, T cells were transfected with either YFP-KCa3.1 or GFP-Kv1.3. The transfected cells were then plated on ICAM-1 surface and allowed to migrate for 2 hr at 37°C. Cells were then fixed and stained with antibodies against markers for the uropod (CD44; Fig. 4A) and the leading-edge (CXCR-4; Fig. 4B) [40]. We observed that KCa3.1 localized in the uropod and displayed a strong correlation with the uropod marker CD44 (0.81±0.03; n = 15), while Kv1.3 showed no correlation with CD44 (0.10±0.01; n = 16) as determined by the Pearson’s correlation coefficient (Fig. 4A and C). On the contrary, Kv1.3 localized in the leading-edge and had a strong correlation with the leading-edge marker CXCR-4 (0.82±0.02; n = 11), whereas KCa3.1 did not (0.07±0.09; n = 8) (Fig. 4B and C). These results indicated that Kv1.3 and KCa3.1 differentially polarize in moving T cells, with Kv1.3 concentrating in the leading-edge and KCa3.1 in the uropod.

The membrane distribution of KCa3.1 at the uropod was confirmed using a HA-tagged KCa3.1 channel. The HA tag is present in the extracellular loop of the KCa3.1 protein, between S3 and S4, thus allowing the visualization of membrane KCa3.1 channels. These channels display electrophysiological properties comparable to wild-type KCa3.1 channels [20]. The membrane localization of KCa3.1 and the uropod together with the uropod marker CD44 in a polarized T cell is shown in the supplemental figure S4. The membrane localization of the KCa3.1 was confirmed by analyzing the image in the X–Z and Y–Z planes, which show absence of intracellular staining of KCa3.1 channels (Fig. S4B).

The distribution of Kv1.3 channels at the leading edge was confirmed for native channels by taking advantage of specific Kv1.3 antibodies [28]. Unfortunately, similar experiments were not possible for KCa3.1 due to the lack of commercially available specific antibodies. The confocal images in figure 4D showed a typical punctate distribution of native Kv1.3, as previously described, and a co-localization of Kv1.3 with CXCR-4 [28]. The Pearson’s correlation coefficient showed a significantly higher correlation of Kv1.3 with the leading-edge marker than with the uropod’s (Fig. 4E). The correlation coefficient of Kv1.3 and CXCR-4 was 0.45±0.07 (n = 9) compared to 0.21±0.03 (n = 11) for Kv1.3 and CD44. This finding confirmed the preferential distribution of Kv1.3 to the leading-edge.

Overall these results indicated that KCa3.1 channels located in the uropod of a migrating cell were significantly involved in the T cell migration machinery, while Kv1.3 channels, localized predominantly in the leading-edge, had no effect on T cell migration.

Since it is known that cell migration is a Ca^2+^-dependent process and KCa3.1 channels are activated by high intracellular Ca^2+^ concentrations ([Ca^2+^]_i_), we proceeded to determine if there is a difference in [Ca^2+^]_i_ in the different compartments of migrating cells.

### Oscillations in [Ca^2+^]_i_ Occur at the Uropod

**Figure 5:**
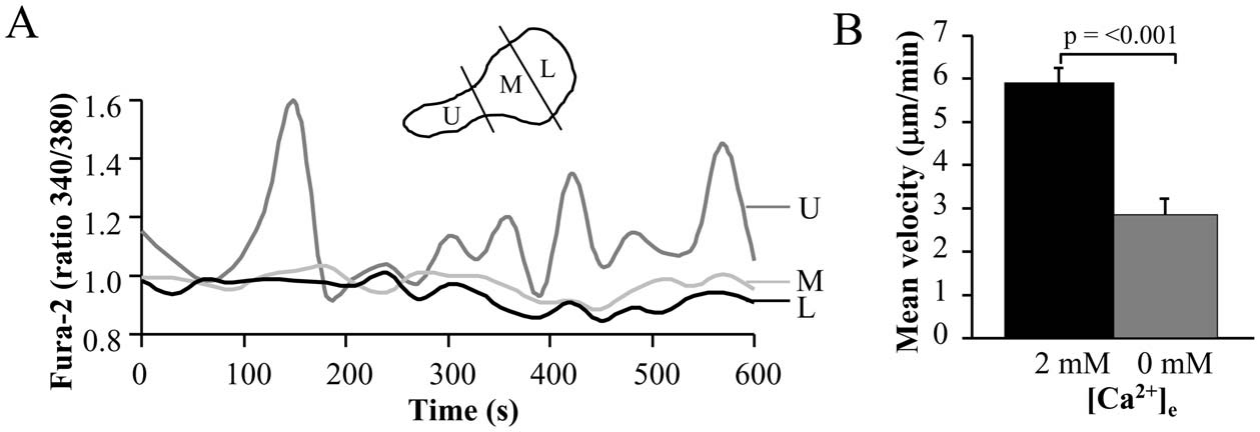
Intracellular Ca^2+^ concentrations and the effect of extracellular Ca^2+^ in migrating activated T cells. A. Time-dependent intracellular Ca^2+^ levels in the uropod (U), mid-body (M) and leading-edge (L) in a representative migrating T cell. Cells were loaded with 1 µM Fura-2, and the Fura-2 340/380 ratio was quantified using the MetaMorph software. A scheme representing the different cell compartments of a polarized migrating cell is shown as inset. B. Effect of extracellular Ca^2+^ removal on T cell migration. The motility was determined by following the same cell in physiological [Ca^2+^]_e_ (2 mM) and in Ca^2+^-free medium (0 Ca^2+^/EGTA). Control motility is the average motility over 20 min, while the motility in Ca^2+^ -free medium corresponds to the average of the last 5 min of 25 min in Ca^2+^-free medium. The data are the average of n = 17 cells from 2 different donors.

The ratiometric fluorescent dye Fura-2 was used to measure the [Ca^2+^]_i_ in the uropod, mid-body and leading-edge of migrating CD3^+^ cells (Fig. 5A). We observed [Ca^2+^]_i_ oscillations in the uropod in 9/19 cells. These oscillations had an average frequency of 0.5±0.1 min^−1^ and a peak [Ca^2+^]_i_ amplitude of 0.33±0.06 r.u. (n = 9) with maximum oscillation up to 0.61 r.u. We did not observe a significant difference in the velocities of migrating cells with [Ca^2+^]_i_ oscillations in the uropod (2.4±0.3 µm/min, n = 9 out of 19 total cells analyzed) compared to the rest of the migrating cell population that did not demonstrate [Ca^2+^]_i_ oscillations in the uropod (2.5±0.2 µm/min, n = 10 out of 19 total cells, p = 0.9). Oscillations with a frequency and peak [Ca^2+^]_i_ amplitude comparable to the oscillations at the uropod (0.37±0.05 min^−1^, p = 0.20 and 0.35±0.12 r.u, p = 0.86, respectively) were also seen at the leading- edge but only in 4/19 cells. Only 50% of the front oscillating cells displayed oscillations at the uropod. The increased occurrence of oscillations at the uropod suggests that higher [Ca^2+^]_i_ may be achieved at the uropod and that the [Ca^2+^]_i_ oscillations could be part of the migration regulatory mechanism that includes KCa3.1. Although the number of cells analyzed is limited to draw any firm conclusions, it appears that the Ca^2+^ oscillations at the uropod may not correlate with the speed of a cell. Still we cannot exclude that the velocity may be dependent on the frequency and/or amplitude of oscillations or that the oscillations may be associated with other features of the movement such as kinetics (i.e. stop and go) and directionality. Further Ca^2+^ studies are necessary to delineate the profile of Ca^2+^ in the different cell compartments.

Further Fura-2 experiments indicated that influx of Ca^2+^ is important for T cell migration as migration was significantly reduced by removal of extracellular Ca^2+^ (Fig. 5B). The cell motility decreased significantly from 5.9±0.3 µm/min in 2 mM extracellular [Ca^2+^] ([Ca^2+^]_e_) to 3.4±0.2 µm/min in 0 mM [Ca^2+^]_e_ (n = 17). This result indicated a significant role of extracellular Ca^2+^ in T cell migration, but also showed that intracellular Ca^2+^ participates in the regulation of migration as the cell motility was not completely abolished in 0 mM [Ca^2+^]_e_. Still, these experiments do not exclude that alterations in Ca^2+^ export and adhesion may contribute to the reduced velocity.

The CRAC channel is a known regulator of Ca^2+^ influx in T lymphocytes [8]. Another channel which could possibly play a role in the Ca^2+^ influx during migration is TRPM7 [15]. The distributions of these channels during T cell migration and their role in the migration process are not fully understood.

### Orai1 is Localized in the Leading-edge Whereas TRPM7 is Located in the Uropod

Confocal experiments were performed to determine the distribution of Orai1 (the pore-forming subunit of CRAC) and TRPM7 in migrating activated T cells [8].

**Figure 6:**
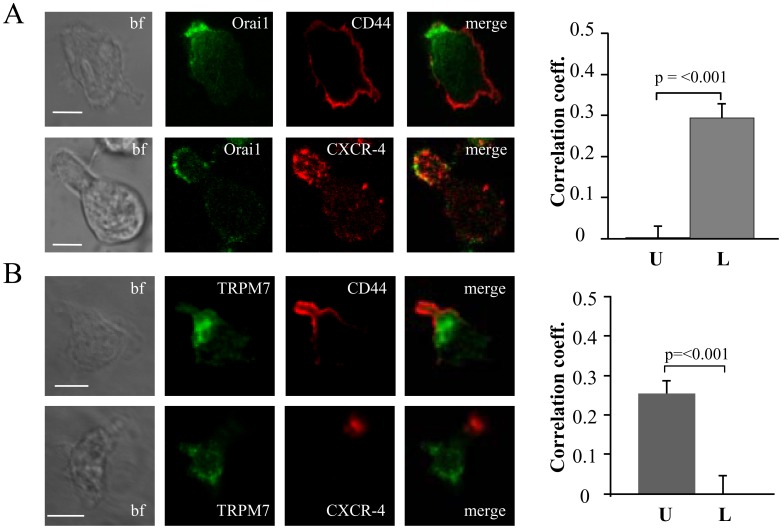
Differential distribution of Orai1 and TRPM7. A. Confocal images of migrating activated T cells stained for Orai1 (green) together with either CD44 (red) or CXCR-4 (red). Yellow areas in the merge images indicate colocalization. Scale bar  = 5 µm. The average correlation coefficients for the uropod (U, n = 21) and the leading-edge (L, n = 22) from 2 donors are shown in the right panel. B. Confocal images of migrating activated T cells that were fixed and stained for TRPM7 (green) together with either CD44 (red) or CXCR-4 (red). Correlation between two proteins is indicated by yellow areas in the merge images. Scale bar  = 5 µm. The average correlation coefficients for the uropod (U, n = 29) and the leading-edge (L, n = 12) from 2 donors are shown in the right panel.

The distribution and localization of Orai1 is shown in Fig. 6A. The images indicated that Orai1 was located in the leading-edge together with the leading-edge marker CXCR-4, and not in the uropod. The Pearson’s correlation coefficient showed that Orai1 had a stronger correlation with CXCR-4 (0.29±0.03; n = 22) and no correlation with CD44 (0.00±0.03, n = 21). On the contrary, TRPM7 localized more in the uropod than in the leading-edge as indicated by a positive correlation with CD44 (0.25±0.03, n = 29) and not with CXCR-4 (0.00±0.05, n = 12) (Fig. 6B). This is characteristic of TRPM7 as another member of the TRPM family, TRPM4 (a Ca^2+^-activated nonselective monovalent cation channel, which acts as a regulator of Ca^2+^ oscillations by depolarizing the membrane with monovalent cations like Na^+^) preferentially localized in the leading-edge (Fig. S5A) [41]. The correlation coefficient for TRPM4 channels with CXCR-4 was 0.28±0.05 (n = 18) compared to −0.18±0.03 (n = 31) with anti-CD44.

Overall, these results indicated that TRPM7 localized together with KCa3.1 at the uropod. The leading-edge instead contained Kv1.3, CRAC and TRPM4. Since KCa3.1, and not Kv1.3, played a crucial role in T cell migration, TRPM7 is a good candidate for increasing [Ca^2+^]_i_ locally at the uropod thus supporting KCa3.1 in its migratory role. Thus, we proceeded to establish the role of CRAC, TRPM7 and TRPM4 in T cell migration.

### TRPM7 Channels are Involved in T Cell Migration

Earlier publications described a role in cell migration for TRPM7 in fibroblasts and TRPM4 in dendritic cells [13], [41]. A migratory role for CRAC was previously shown in vascular smooth muscle cells and in dendritic cells [11], [12]. To determine if any of these channels is also involved in the migration of activated CD3^+^ T cells we used blockers against these channels. We determined the mean velocity by following single cells before and after application of various inhibitors.

**Figure 7:**
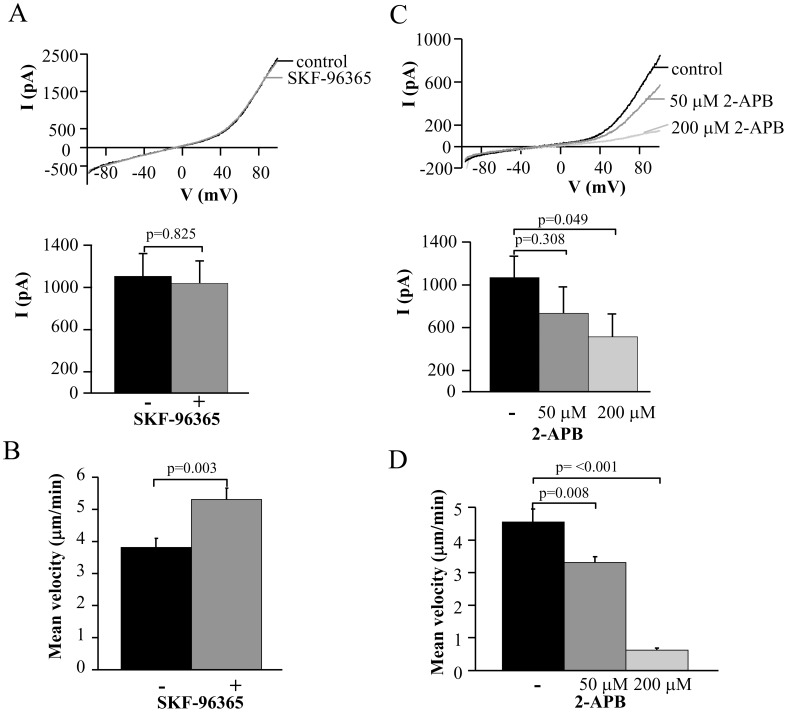
Selective inhibitory effect of TRPM7 blockade on activated T cell migration. A. Lack of effect of SKF-96365 on TRPM7 currents. Representative TRPM7 currents in activated T cells were recorded before (control) and after extracellular application of 20 µM SKF-96365. Currents were induced from a HP of 0 mV by ramp depolarization from −100 mV to +100 mV every 10 s. The average current amplitude (lower panel) for TRPM7 channels was calculated at +100 mV (n = 8). B. Effect of SKF-96365 (20 µM) on T cell migration. T cell migration was measured in the same cell before and after treatment with the blocker. The data are the average of 18 cells from 2 individuals. C. Inhibition of native TRPM7 currents by 2-APB. Representative TRPM7 currents in activated T cells were recorded before (control) and after increasing concentrations of 2-APB. Currents were elicited like in A. The corresponding average current amplitudes are shown in the bottom panel (n = 7). D. Effect of 2-APB on T cell migration. T cell migration was measured in the same cell before and after treatment with progressive concentrations of 2-APB. The data are the mean of 15 cells from 2 individuals. Statistical significance in panels C and D was determined by One Way Repeated Measures ANOVA and post-hoc testing for significance was determined by Holm-Sidak method. * represents statistical significance in the treatment groups as compared to the control.

Inhibition of CRAC channels was induced by 20 µM SKF-96365. It was previously shown that at this concentration SKF-96365 is a potent inhibitor of CRAC channels, but it does not inhibit TRPM7 channels [42]. To confirm that 20 µM SKF-96365 had no inhibitory effect on TRPM7 channels we performed electrophysiological studies (Fig. 7A). TRPM7 currents recorded before and after addition of SKF-96365 had similar amplitudes: 1107±212 pA and 1040±209 pA (n = 8), respectively. SKF-96365 did not inhibit the mean velocity in migrating activated CD3^+^ T cells, but, surprisingly, increased it 1.4 fold (n = 18) (Fig. 7A). The cells were migrating with a mean velocity of 3.8±0.3 µm/min before SKF-96365 and 5.3±0.4 µm/min after SKF-96365 addition (n = 18).

Next, we used 2-Aminoethoxydiphenyl borate (2-APB). Although, to this date, a specific blocker of TRPM7 channels is unknown, 2-APB has been used to study TRPM7 channels [14], [43]. 2-APB is a known modulator of CRAC channels that produces full inhibition of these channels in T cells at a concentration of 50 µM, but at higher concentrations, also inhibits TRPM7 channels [18], [42], [43]. Indeed, electrophysiological experiments showed that 2-APB inhibits TRPM7 currents in a dose-dependent manner (Fig. 7C). The control current amplitude was reduced from 1070±198 pA to 735±245 at 50 µM and to 512±215 pA at 200 µM 2-APB (Fig. 7C). Similarly, exposure to 50 µM and 200 µM 2-APB reduced the mean velocity of activated CD3^+^ T cells from 4.5±0.4 µm/min to 3.3±0.2 µm/min and 0.6±0.1 µm/min, respectively (Fig. 7D).

**Figure 8:**
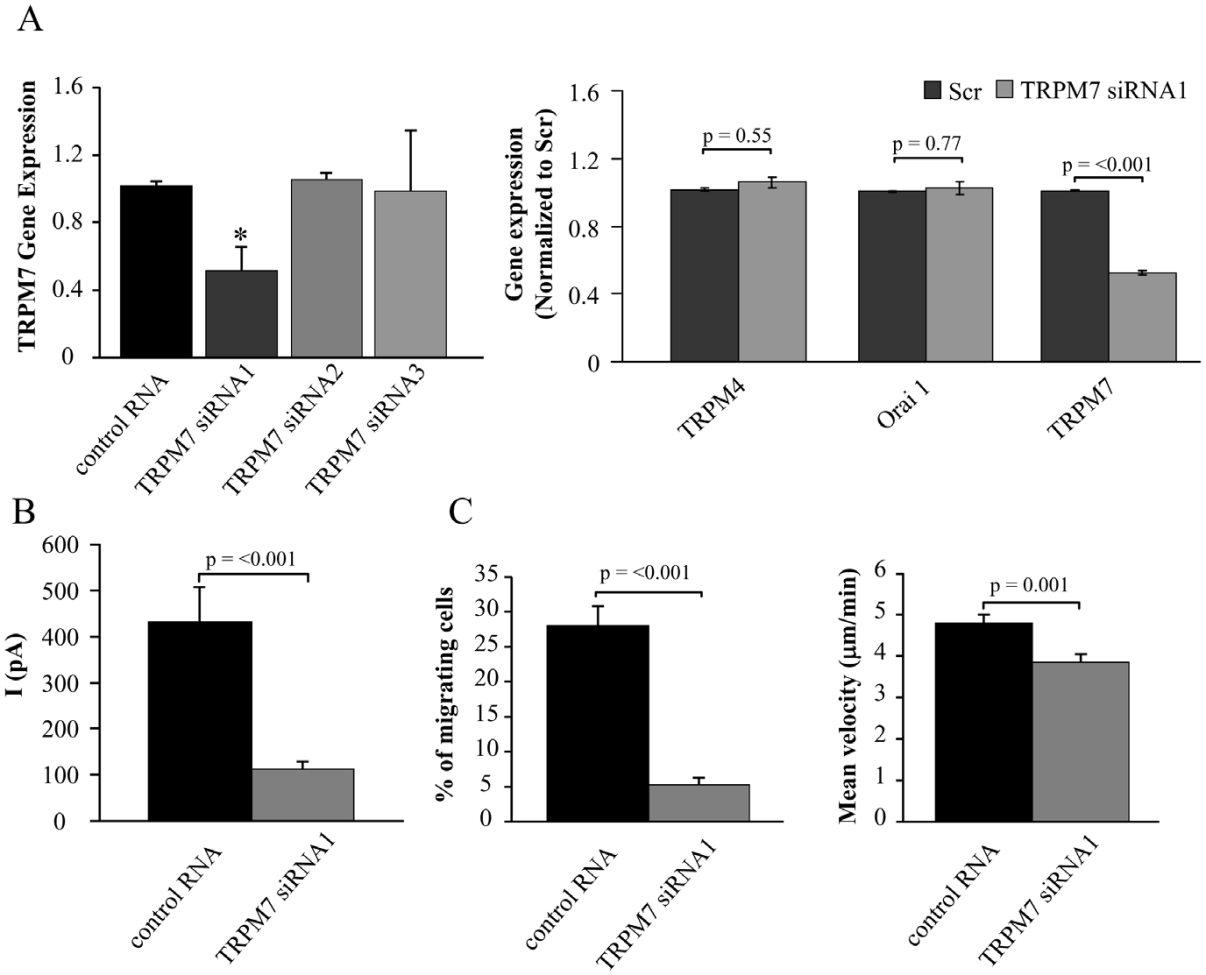
TRPM7 channels are crucial for T cell migration. A. Left Panel: TRPM7 gene expression is reduced by siRNA1. TRPM7 gene expression was quantified by RT-qPCR and is given in fold change relative to GAPDH expression. The data are normalized to control RNA transfected cells and correspond to mean ± SEM of 3 healthy donors each in quadruplicate. Statistical significance was determined by One Way ANOVA, while post-hoc testing was done by Holm-Sidak method. * indicates statistical significance. Right Panel: Specificity of TRPM7 siRNA. Activated T cells were transfected with TRPM7 siRNA1 for 48 hours and the gene expression for TRPM4, Orai1 and TRPM7 was quantified by RT-qPCR. The data are shown as fold change in gene expression relative to GAPDH and are normalized to control RNA transfected cells. Data are presented as mean ± SEM for 3 independent donors, with samples in quadruplicate. B. TRPM7 knockdown decreases TRPM7 currents. The average TRPM7 current was significantly decreased in TRPM7 siRNA1 transfected cells (n = 13) compared to cells transected with control RNA (n = 10). Transected cells were visualized by GFP expression. C. Effect of TRPM7 siRNA1 on T cell migration. On the left panel is reported the % of cells migrating. The total number of cells were for control RNA 626 (n = 5 experiments; 2 donors) and for TRPM7 siRNA1 796 (n = 8 experiments; 2 donors). Right panel: T cell migration was measured in TRPM7 siRNA1 transfected cells (n = 40) and compared to control RNA transfected cells (n = 49) from 2 individuals.

To confirm that TRPM7 channels are essential for T cell migration we used siRNAs to suppress TRPM7 expression. RT-qPCR experiments with 3 different siRNAs against TRPM7 showed that TRPM7 siRNA1 significantly reduced the gene expression of TRPM7 in primary activated T cells (Fig. 8A, left panel). If we consider a 50% transfection efficiency as reported by us previously, this degree of inhibition in the mixed population of cells used for RT-qPCR translates into a substantial decrease in TRPM7 expression in transected cells [22]. Furthermore, this siRNA was specific for TRPM7 as the gene expression of Orai1 and TRPM4 remained unchanged (Fig. 8A, Right Panel). Due to the limited amount of primary T cells we could not perform the quantification of the protein expression by western blot. However, a decrease in protein expression was measured in the patch-clamp experiments. Since the cells were transfected with siRNAs and a GFP plasmid, only the transfected cells, recognized by the GFP fluorescence, underwent patch-clamping. The average current in TRPM7 siRNA1 transfected CD3 cells was 112±16 pA (n = 13; 2 donors) compared to 431±75 pA (n = 10; 2 donors) in control cells transfected with control RNA (Fig. 8B). The cell capacitance was not significantly changed, 4.8±0.4 pF (n = 13; 2 donors) in TRPM7 siRNA1 and 5.4±0.4 pF (n = 10; 2 donors; p = 0.34) in control cells. We thus used TRPM7 siRNA1 to study the effect of TRPM7 downregulation on T cell migration.

Downregulation of TRPM7 channels by siRNA1 significantly reduced the number of migrating cells (Fig. 8C). In control RNA transfected cells we observed almost 30% of cells migrating whereas in siRNA1 transfected cells the migrating cells were 5% of the total number of cells in the field (Fig. 8C). Furthermore, the mean velocity of the migrating cells was significantly decreased in TRPM7 siRNA-treated compared to control RNA transfected T cells (Fig. 8C). The cells were migrating with a mean velocity of 4.8±0.2 µm/min in control RNA cells (n = 49) and 3.8±0.2 µm/min in TRPM7 siRNA1 cells (n = 40). These effects can be explained taking into account that the majority of the transected cells had an almost complete knock down of TRPM7 which led to lack of motility and is reflected by the significant reduction in the % of migrating cells. Other cells that may have had a partial TRPM7 downregulation were still migrating, but with a lower velocity.

These findings indicated that TRPM7, and not CRAC, facilitates the migration of activated T cells. Furthermore, we excluded TRPM4 from having a role in T cell migration as its expression is not altered by TRPM7 siRNAs. Furthermore, glibenclamide (100 µM), a non-specific inhibitor of TRPM4 channels, did not have any effect on T cell migration (Fig. S5B) [44]. The mean velocity of activated T cells after glibenclamide (3.9±0.3 µm/min) was comparable to the migration before glibenclamide addition (3.4±0.2 µm/min, n = 16).

Overall, these results provide evidence of a role of TRPM7 and KCa3.1 at the uropod in T cell migration.

## Discussion

In the present study we demonstrated the differential compartmentalization and functional role of ion channels in migrating activated human T cells. KCa3.1 and TRPM7 localize in the uropod and control the cell migration velocity, while Kv1.3, CRAC and TRPM4 accumulate in the cell front and do not facilitate cell motility.

The polarization and motility of activated T cells was studied in-vitro using ICAM-1 surfaces. Similar surfaces were previously used as artificial substrates to study T cell polarization and formation of the immunological synapse (IS) [45], [46]. These ICAM-1 surfaces were proven to be ideal for T cell migration studies since T cells crawling on them displayed high cell adhesion together with high cell motility [16]. We observed that activated human T cells migrated on ICAM-1 surfaces with a mean velocity of 2–6 µm/min, which is comparable to what was observed in other in-vitro studies [34], [45], [47]. Donor to donor variability can account for the differences in mean velocity between our experiments. Higher velocities were reported in in-vivo studies. T cells in rat lymph nodes migrated with a mean velocity of ∼16 µm/min, whereas the average velocity of T cells in mouse lymph node was about 10–12 µm/min [10]. Difference in species, activation states and lack of chemoattractant in the in-vitro experiments may account for the differences with the in-vivo studies.

While many aspects of T cell mobility have been thoroughly studied, the role of ion channels is not yet defined. Although all the main ion channels expressed in human T cells have been shown to be involved in the motility of other cell types, we discovered that only KCa3.1 and TRPM7, both localized at the uropod, were crucial for the migration of activated human T cells. A role for KCa3.1 in cell migration was previously described for various cell types including human lung mast, MDCK-F, microglia and coronary smooth muscle cells [5]. In all these cell types KCa3.1 inhibition slowed down cell migration. In this study we showed that KCa3.1 plays a similar role in the motility of activated human T cells. TRPM7 was also involved in the migration process of other cell types. Downregulation of TRPM7 decreased the motility of fibroblasts and tumor cells [13], [14]. TRPM7 channels are expressed in T cells, particularly in activated T cells (from ∼15 channels in resting T cell to ∼140 channels in activated T cell), but their functional role in T cells has not yet been established, although it has been suggested that they may control Mg^2+^ homeostasis [8], [26], [48]. We could demonstrate for the first time a physiological role for TRPM7 channels in T cell migration. Suppression of TRPM7 channels resulted in an overall decreased in number of migrating cells and lower velocity of the migrating cells. Thus, the possibility that TRPM7 regulates T cell migration, which is a crucial part of the immune response, is of great significance and we herein presented evidence that TRPM7 is involved in the migration machinery of activated CD3^+^ T cells.

The role of KCa3.1 and TRPM7 on T cell migration is specific for these channels. Inhibition of the ion channels that accumulate preferentially in the leading-edge of the T cell, namely Kv1.3 and TRPM4, did not influence the cell migration. The role of Kv1.3 in cell migration is not so well defined as for KCa3.1 and it has been shown that inhibition of Kv1.3 decreased the migration velocity in microglia cells, vascular smooth muscle cells, and CD4^+^ T_em_ cells in rats [10], [49], [50]. No effect was reported for rat resting T cells in the lymph nodes [10]. The discrepancy with our finding could be due to the cell type and, for T cells, to the T cell activation state or the animal origin. Rat T_CM_ and T_EM_ effector cells were described to have the same channel pattern like human T cells [8]. However, we observed a difference in the sensitivity to ShK probably due to either the difference between in-vivo and in-vitro recordings or the fact that Matheu and colleagues treated the rats over a long time period (3 to 42 hr) [10]. Furthermore, Kv1.3, and not KCa3.1, constitutes the predominant K^+^ conductance of activated T_em_ cells [8]. In addition, we could exclude a migratory role for TRPM4, which is instead important in dendritic and mast cells, as the cell migration was reduced by silencing TRPM7 with no decrease in TRPM4 expression [41], [51]. Interestingly, SKF-96365, an inhibitor of the other channel that distributes to the leading-edge, CRAC, caused a significant increase in the T cell migration velocity. A similar effect was already reported by Svensson and colleagues [52]. They showed that T cells expressing a non-functional Orai1 mutant (Orai1-R91W) migrated faster than normal T cells. In other cells types it has been instead shown that CRAC inhibition decreases vascular smooth muscle and dendritic cell motility [11]. Still, because SKF-96365 also inhibits TRPC1 channels, which are expressed in primary human T cells, the exact molecular candidate of the increased motility in these experiments remains elusive [53].

Our findings also indicated that the channels that facilitate migration, KCa3.1 and TRPM7, are localized in the uropod while Kv1.3, TRPM4, and CRAC accumulate in the leading-edge. The functional importance of KCa3.1 at the uropod in cell motility was shown previously in migrating polarized epithelial MDCK-F cells [5]. Less is instead known regarding the localization of the other ion channels in polarized migrating cells. In neutrophils, Kv1.3 localized in the lamellipodium which forms the leading-edge [54]. Orai1 was shown to be located at the uropod in human T cells overexpressing Orai1 [55]. To our knowledge, there are no reports about the localization of TRPM7 and TRPM4. Our study is the first report showing the localization of these channels in moving T cells.

Since both KCa3.1 and TRPM7 are located in the cell rear and are crucial for lymphocyte migration, it is likely that they are closely positioned to support each other’s function. KCa3.1 is activated by increased [Ca^2+^]_i_ and TRPM7 could deliver the required amount of Ca^2+^, while KCa3.1 may regulate the electrochemical driving force for Ca^2+^ influx via TRPM7. The concerted function of KCa3.1 and TRPM7 at the uropod could contribute to the oscillatory [Ca^2+^]_i_ observed in this cell compartment. The [Ca^2+^]_i_ oscillations observed in the uropod are consistent with an intracellular Ca^2+^ gradient, with higher [Ca^2+^]_i_ reached at the uropod, which may serve to support the Ca^2+^ requirements of the opposing cellular events occurring at the two poles of a migrating cell. An intracellular Ca^2+^ gradient with a lower concentration in the leading-edge and a higher concentration in the uropod has been reported in many cells including eosinophils, but not in mouse T cells [5], [47].

The position of Kv1.3 and CRAC in the leading-edge could instead have a strategic advantage for a fast immune response. Lymphocyte migration is necessary for scanning of the APCs to recognize specific antigens which is followed by TCR binding to the cognate peptide major histocompatibility complex (pMHC) [2]. The TCR engagement happens at the leading-edge of a polarized migrating cell and leads to formation of the IS [3], [16]. Although it has been shown that Kv1.3, KCa3.1 and CRAC localize in the mature IS, it is possible that only Kv1.3 and CRAC are found in the initial TCR microclusters of the immature IS that are associated with active signaling and the onset of the activation response [19], [28], [55]. We cannot exclude that Kv1.3 may be part of this early IS signaling systems (thus its localization in the leading-edge), while KCa3.1 moves at later time points and regulates the development of Ca^2+^ signaling. Kv1.3 sets the resting membrane potential necessary for the initial Ca^2+^ influx thorough CRAC channels [8], [56]. This could also be the case for TRPM4 which has been shown to modulate Ca^2+^ influx through CRAC channels [41]. Still, there is no report whether TRPM4 can be found in the IS. Furthermore, Kv1.3 associates to β1 integrins and may thus participate in the adhesion of the T cell to the APC [57]. It is tempting to speculate that Kv1.3, CRAC and TRPM4 localization in the leading-edge of a polarized migrating cell could assure a rapid cell-to-cell association and response.

Overall, this study for the first time provides evidence of the selective compartmentalization of ion channels in migrating T cells and introduces a possible role for TRPM7 channels in the immune response.

## Supporting Information

Figure S1 **Optimization of the polymer layer.** A. Light microscopy images of Si/SiO_2_ wafer chips coated with 3% PNMP solution, developed in pH 7.4 with two different time points of UV irradiation using TedPella mask. B. Effect of varying pH levels on the thickness of the PNMP polymer layer. All PNMP layers were coated on using optimized uniform specifications in different % PNMP solution (Table S1) with a UV irradiation for 45 min. C. Light microscope images of Si/SiO_2_ wafer chips coated with 1% PNMP solution and 12 min UV irradiation using TedPella mask and developed in different pH levels. D. Scanning Electron Microscopy (SEM) images of PNMP-pattern.
(TIF)

Figure S2 **Specificity of Orai1, TRPM4 and TRPM7 antibodies.** Activated primary T cells were fixed and stained with Orai1 (top*)*, TRPM4 (middle) and TRPM7 (bottom) antibodies (ab). The corresponding images of cells treated with secondary antibodies only (2° only), no antibodies (cells only) or the antibody preadsorbed to the corresponding antigen (preadsorbed) are shown as right side panels. The corresponding DIC micrographs for each set are shown in the bottom of the florescence images.
(TIF)

Figure S3 **Kv1.3 channels do not affect the cell migration in primary activated CD3^+^ T cells but KCa3.1 channels decreased significantly the velocity.** A. Migration of a representative activated CD3^+^ T cells on ICAM-1, recorded by time-lapse bright-field microscopy, before and after application of ShK (10 nM). Illustrated are snapshots of different time points. The asterisk represents the starting point, and arrow the initial direction. The dotted line is the coming track, and the continuous line is the covered distance. The time-lapse bright-field microscopy was recorded continuously with a gap of 1.5–2 min to add ShK indicated by the arrow at 22 min. B. Representative migrating CD3^+^ T cells before and after application of TRAM-34 (250 nM) in regarding to the time recorded by time-lapse bright-field microscopy. The recording was continuous with a gap of 1.5–2 min for TRAM-34 application at 22 min. Illustrated are snapshots of different time points. The asterisk shows the starting point, whereas the arrow represents the initial direction. The dotted line is the coming track, and the continuous line is the covered distance. Scale bar  = 5 µm.
(TIF)

Figure S4 **HA-KCa3.1 channels accumulation at the uropod.** A. T cells from one healthy donor were transiently transfected with HA-KCa3.1. After 2 h pre-incubation on the array, cells were fixed and stained with anti-HA (green) and anti-CD44 (red) antibodies. Images were obtained by confocal microscopy as described under materials and methods. The polarized cell is marked by the arrow in the brightfield image. Yellow color indicates colocalization of the HA-KCa3.1 and CD44 signals. Scale bar  = 5 µm. B. Membrane localization of the KCa3.1 channels was conformed by analyzing the merged image in Panel A in X–Z and Y–Z planes. The X–Z and Y–Z scans of the images show that KCa3.1 is present at the cell periphery along with CD44, thereby confirming that in the migrating cells membrane KCa3.1 channels accumulate at the uropod of the polarized migrating T cell.
(TIF)

Figure S5 **Native TRPM4 channels are localized in the leading-edge and have no migratory role in activated CD3^+^ T cells.** A. Confocal images of migrating activated CD3^+^ T cells (left) stained for TRPM4 (green) together with anti-CD44 (uropod; red) and anti-CXCR-4 (leading-edge; red) antibodies. Bright-field (bf) images are in the left panels and merge images are in the right panels. Yellow areas indicate colocalization. Scale bar  = 5 µm. The correlation coefficient (right) indicates that TRPM4 channels are localized in the leading-edge (n = 18) and not in the uropod (n = 31). B. The effect of 100 uM glibenclamide was obtained by following single cells by time-lapse bright-field microscopy before and after treatment with the blocker. The mean velocity shows no significant change in cell migration after inhibition of TRPM4 channels (n = 16).
(TIF)

Table S1 **PNMP uniform spin-coating specifications.**
(TIF)
