# Supplementary figures and images for: KCa3.1 and TRPM7 Channels at the Uropod Regulate Migration of Activated Human T Cells
Source: PLoS One. 2012 Aug 27;7(8):e43859. doi: 10.1371/journal.pone.0043859 (PMC3428288; doi:10.1371/journal.pone.0043859)

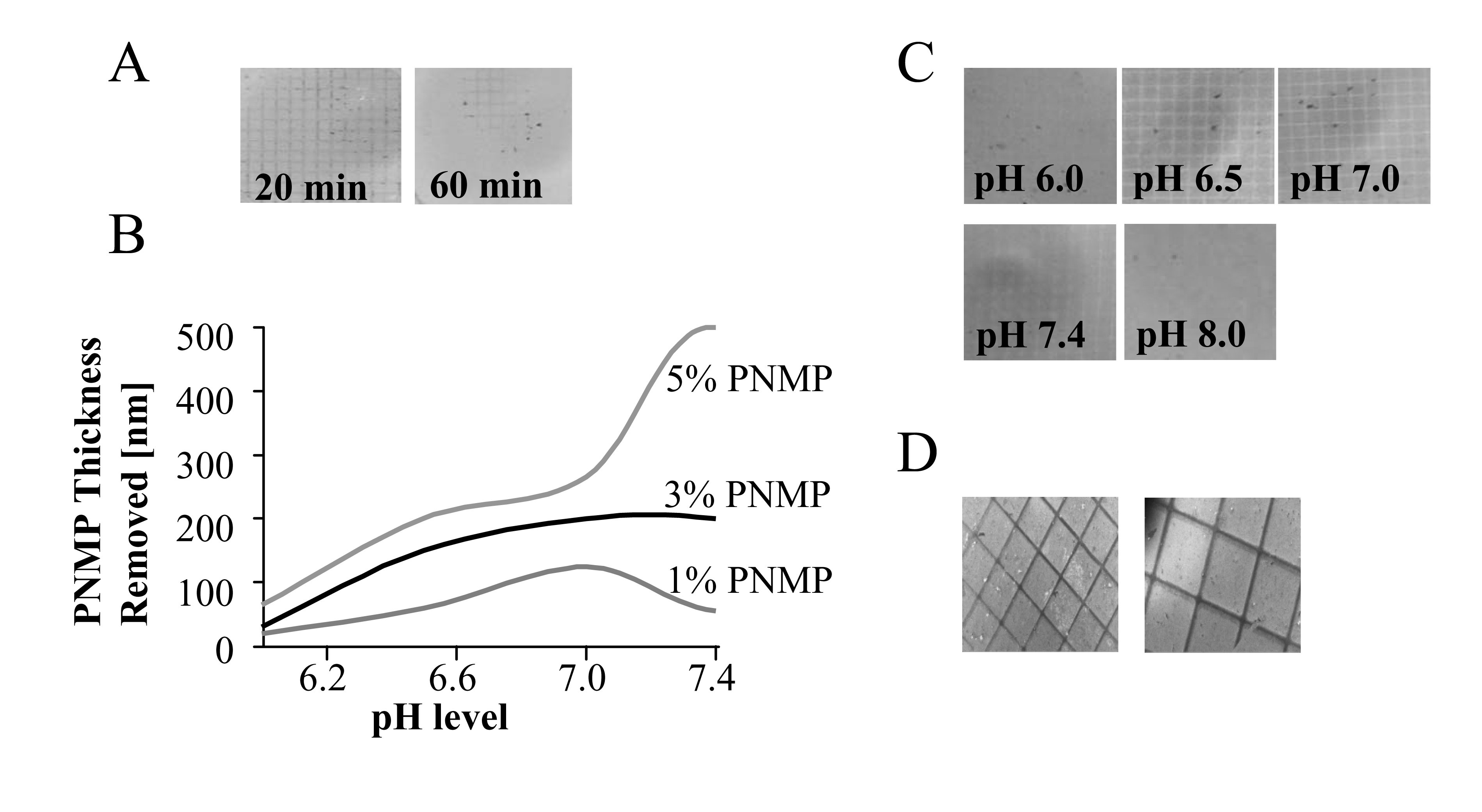

Supplement: Figure S1 — Optimization of the polymer layer. A. Light microscopy images of Si/SiO2 wafer chips coated with 3% PNMP solution, developed in pH 7.4 with two different time points of UV irradiation using TedPella mask. B. Effect of varying pH levels on the thickness of the PNMP polymer layer. All PNMP layers were coated on using optimized uniform specifications in different % PNMP solution (Table S1) with a UV irradiation for 45 min. C. Light microscope images of Si/SiO2 wafer chips coated with 1% PNMP solution and 12 min UV irradiation using TedPella mask and developed in different pH levels. D. Scanning Electron Microscopy (SEM) images of PNMP-pattern. (TIF) [file pone.0043859.s001.tif]

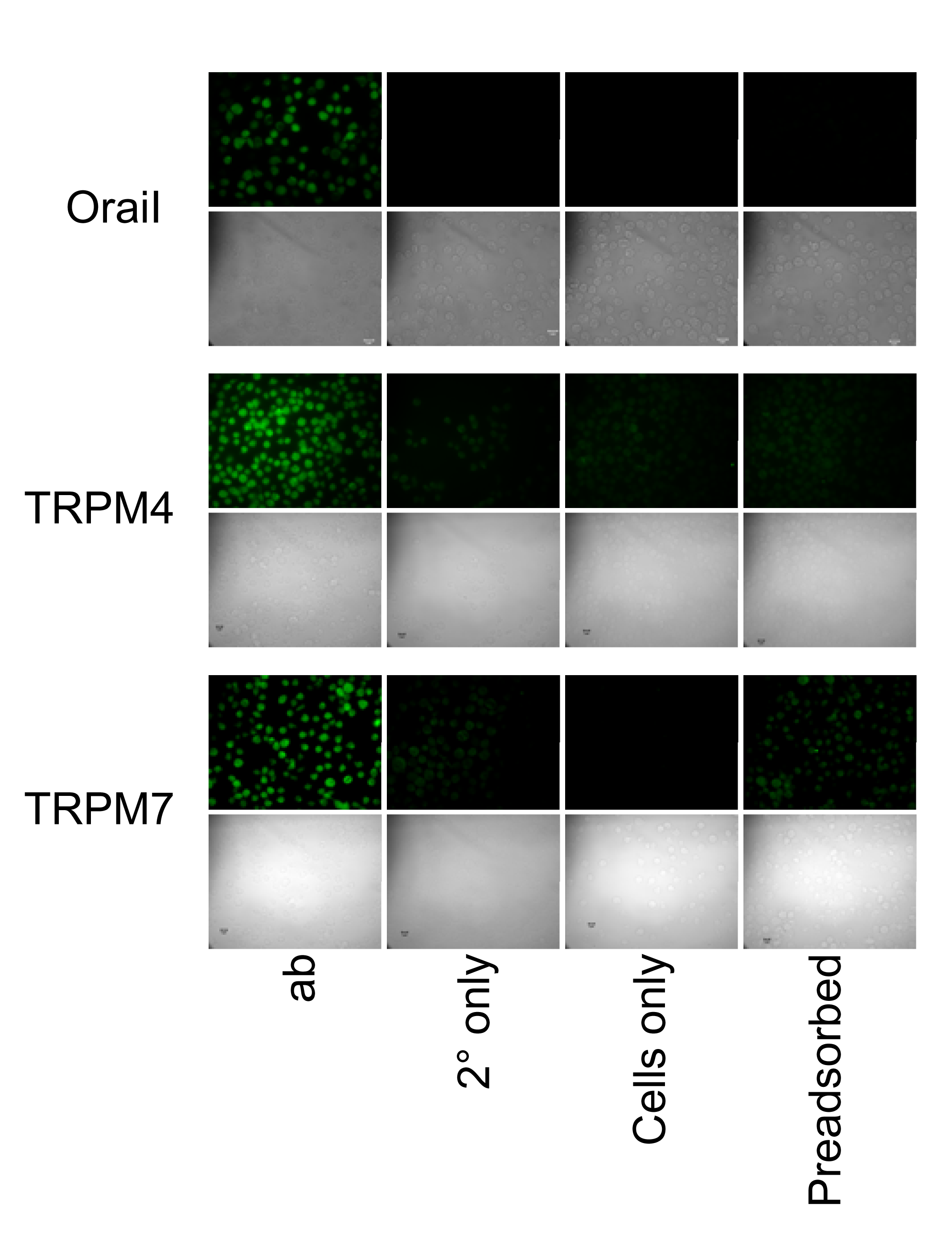

Supplement: Figure S2 — Specificity of Orai1, TRPM4 and TRPM7 antibodies. Activated primary T cells were fixed and stained with Orai1 (top), TRPM4 (middle) and TRPM7 (bottom) antibodies (ab). The corresponding images of cells treated with secondary antibodies only (2° only), no antibodies (cells only) or the antibody preadsorbed to the corresponding antigen (preadsorbed) are shown as right side panels. The corresponding DIC micrographs for each set are shown in the bottom of the florescence images. (TIF) [file pone.0043859.s002.tif]

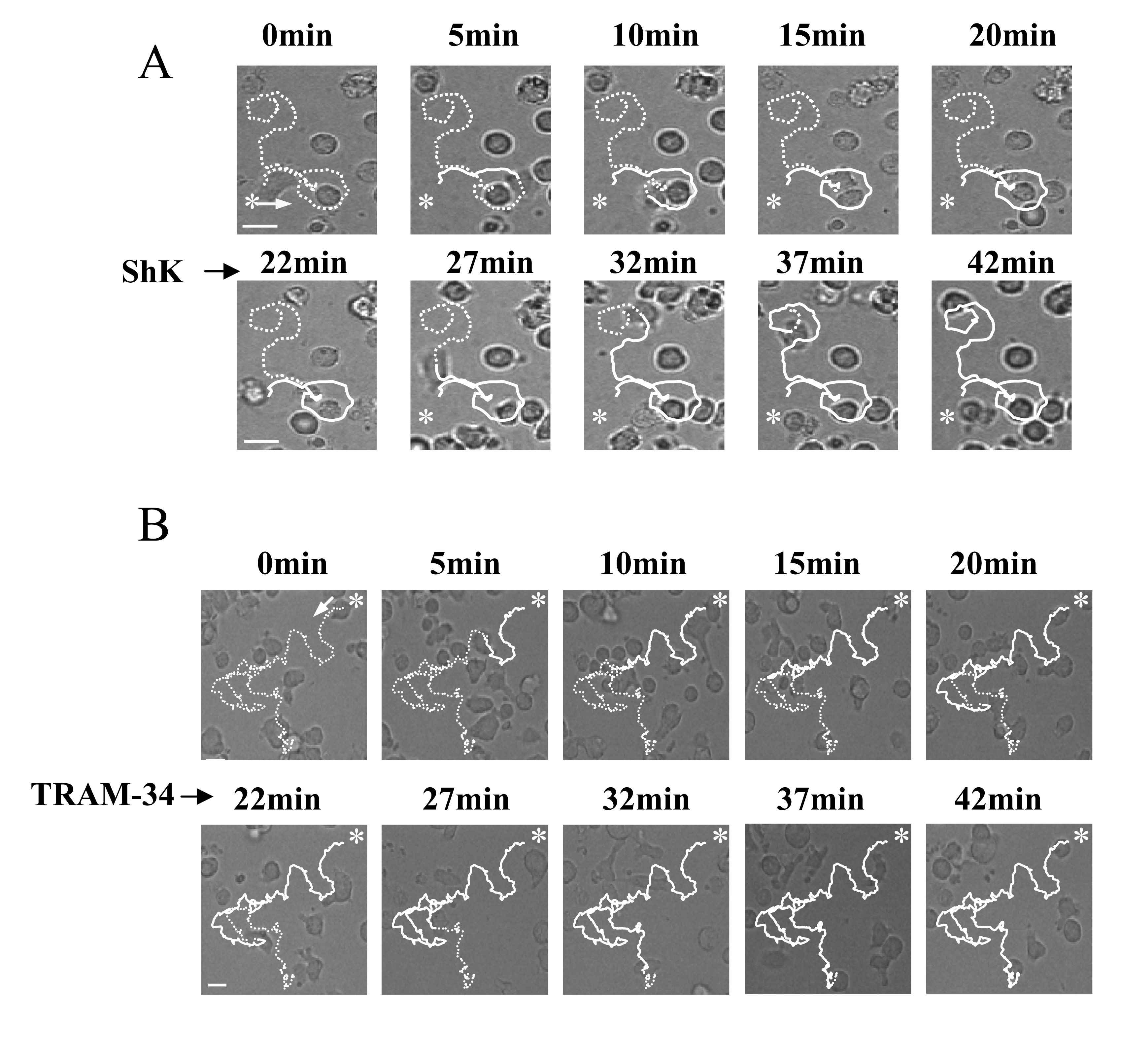

Supplement: Figure S3 — Kv1.3 channels do not affect the cell migration in primary activated CD3+ T cells but KCa3.1 channels decreased significantly the velocity. A. Migration of a representative activated CD3+ T cells on ICAM-1, recorded by time-lapse bright-field microscopy, before and after application of ShK (10 nM). Illustrated are snapshots of different time points. The asterisk represents the starting point, and arrow the initial direction. The dotted line is the coming track, and the continuous line is the covered distance. The time-lapse bright-field microscopy was recorded continuously with a gap of 1.5–2 min to add ShK indicated by the arrow at 22 min. B. Representative migrating CD3+ T cells before and after application of TRAM-34 (250 nM) in regarding to the time recorded by time-lapse bright-field microscopy. The recording was continuous with a gap of 1.5–2 min for TRAM-34 application at 22 min. Illustrated are snapshots of different time points. The asterisk shows the starting point, whereas the arrow represents the initial direction. The dotted line is the coming track, and the continuous line is the covered distance. Scale bar = 5 µm. (TIF) [file pone.0043859.s003.tif]

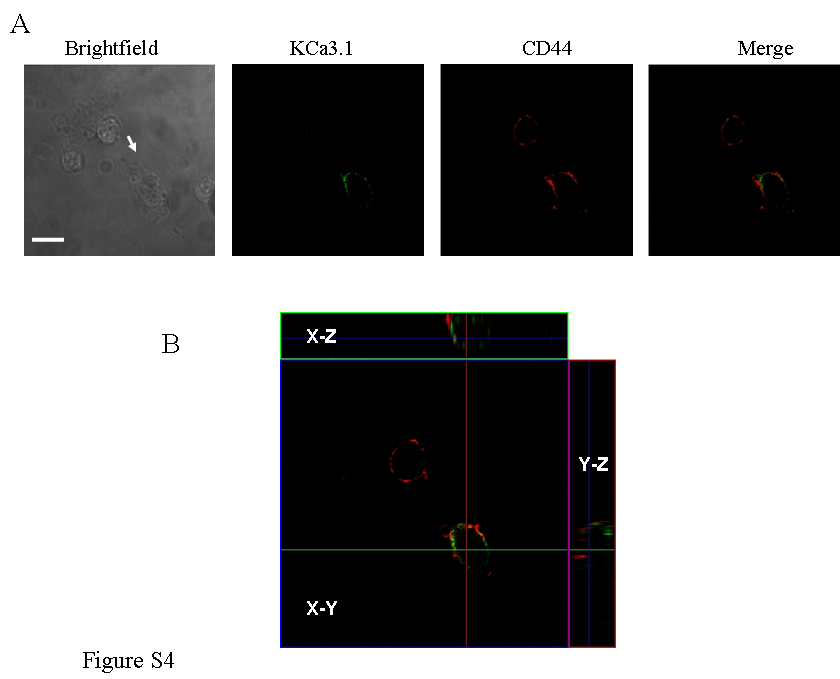

Supplement: Figure S4 — HA-KCa3.1 channels accumulation at the uropod. A. T cells from one healthy donor were transiently transfected with HA-KCa3.1. After 2 h pre-incubation on the array, cells were fixed and stained with anti-HA (green) and anti-CD44 (red) antibodies. Images were obtained by confocal microscopy as described under materials and methods. The polarized cell is marked by the arrow in the brightfield image. Yellow color indicates colocalization of the HA-KCa3.1 and CD44 signals. Scale bar = 5 µm. B. Membrane localization of the KCa3.1 channels was conformed by analyzing the merged image in Panel A in X–Z and Y–Z planes. The X–Z and Y–Z scans of the images show that KCa3.1 is present at the cell periphery along with CD44, thereby confirming that in the migrating cells membrane KCa3.1 channels accumulate at the uropod of the polarized migrating T cell. (TIF) [file pone.0043859.s004.tif]

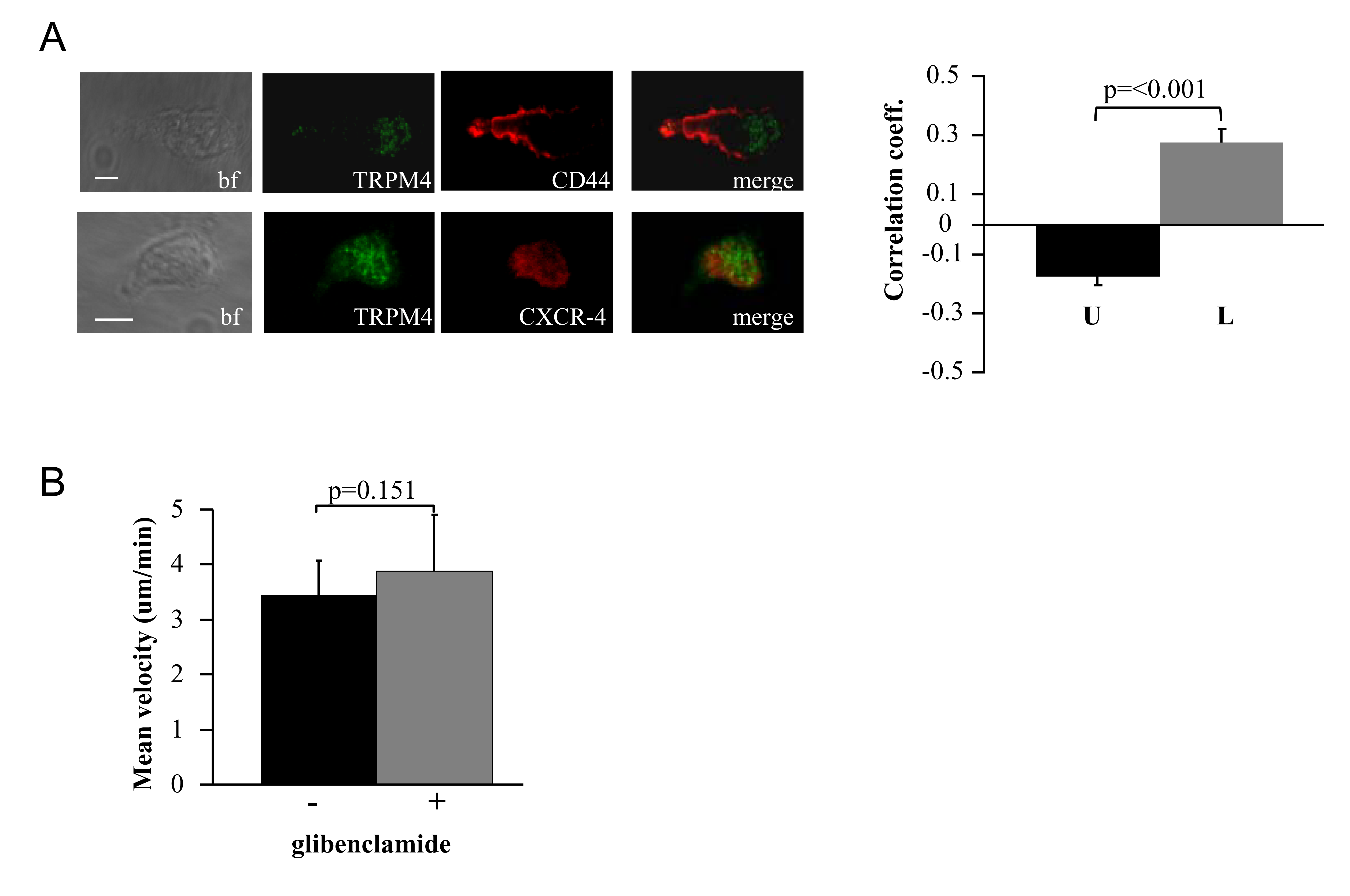

Supplement: Figure S5 — Native TRPM4 channels are localized in the leading-edge and have no migratory role in activated CD3+ T cells. A. Confocal images of migrating activated CD3+ T cells (left) stained for TRPM4 (green) together with anti-CD44 (uropod; red) and anti-CXCR-4 (leading-edge; red) antibodies. Bright-field (bf) images are in the left panels and merge images are in the right panels. Yellow areas indicate colocalization. Scale bar = 5 µm. The correlation coefficient (right) indicates that TRPM4 channels are localized in the leading-edge (n = 18) and not in the uropod (n = 31). B. The effect of 100 uM glibenclamide was obtained by following single cells by time-lapse bright-field microscopy before and after treatment with the blocker. The mean velocity shows no significant change in cell migration after inhibition of TRPM4 channels (n = 16). (TIF) [file pone.0043859.s005.tif]

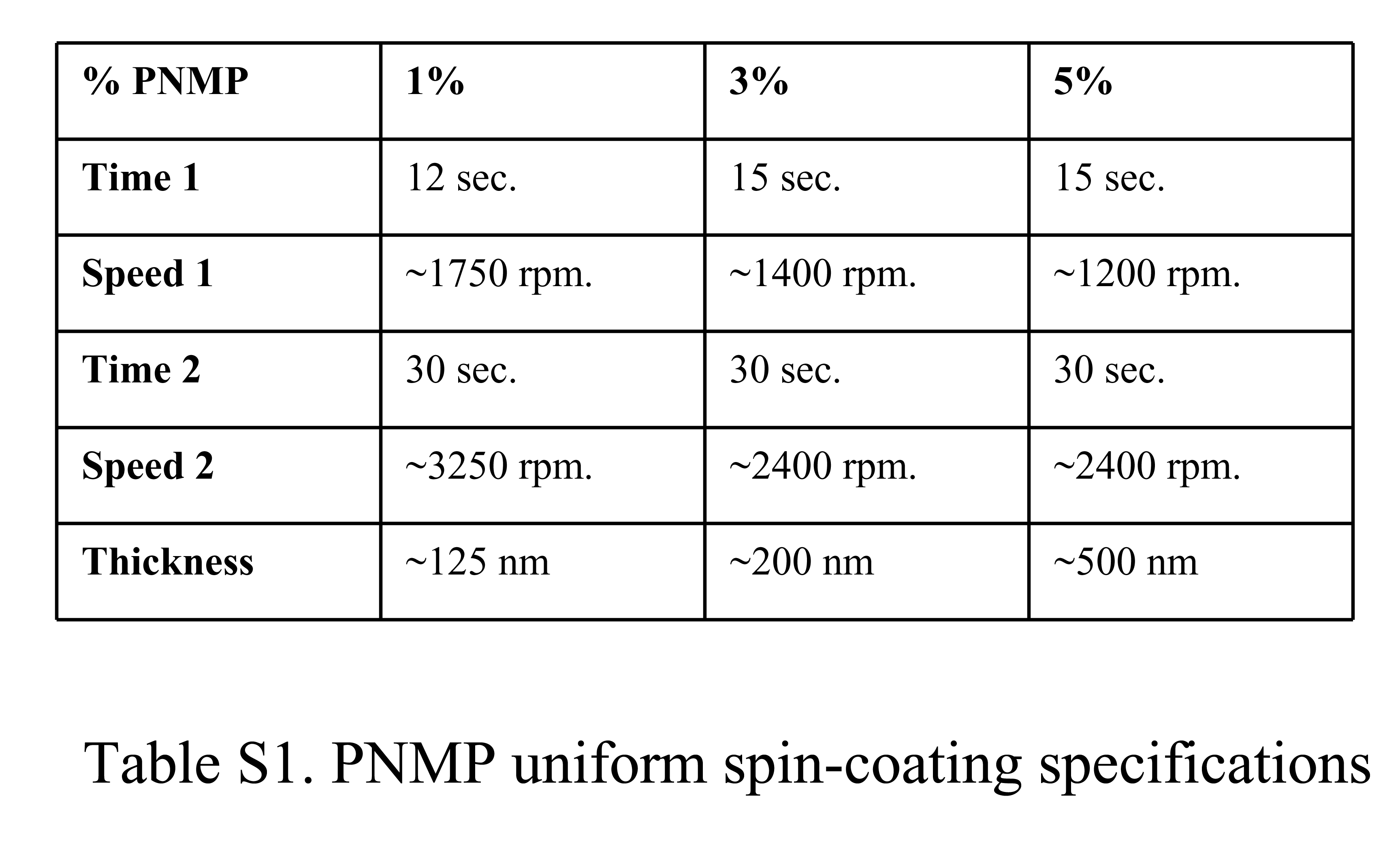

Supplement: Table S1 — PNMP uniform spin-coating specifications. (TIF) [file pone.0043859.s006.tif]
